# Carbon Nanotubes and Carbon Nanotube Structures Used for Temperature Measurement

**DOI:** 10.3390/s19112464

**Published:** 2019-05-29

**Authors:** Bogdan Florian Monea, Eusebiu Ilarian Ionete, Stefan Ionut Spiridon, Daniela Ion-Ebrasu, Emil Petre

**Affiliations:** 1 National R&D Institute for Cryogenics and Isotopic Technologies ICSI Rm. Valcea, Ramnicu Valcea 240050, Romania; bogdan.monea@icsi.ro (B.F.M.); ionut.spiridon@icsi.ro (S.I.S.); daniela.ebrasu@icsi.ro (D.I.-E.); 2 Faculty of Automation, Computers and Electronics, University of Craiova, Craiova 200440, Romania; epetre@automation.ucv.ro

**Keywords:** carbon nanotubes, temperature sensor, CNT assembling, sensitivity, film, polymer, nanocomposite

## Abstract

Accurate measurement of temperatures with low power consumption with the highest sensitivity and smallest possible elements is still a challenge. The thermal, electrical, and mechanical properties of carbon nanotubes (CNTs) have suggested that their use as a very sensitive sensing element will allow the creation of different sensors, far superior to other devices of similar size. In this paper, we present a short review of different constructive designs of CNTs based resistive sensors used for temperature measurement, available in literature, assembled using different processes, such as self-assembly, drop-casting from a solution, thin films obtained by gluing, printing, spraying, or filtration over a special membrane. As particular cases, temperature sensors obtained from CNT-polymer nanocomposite structures, CNTs filled with uniformly dispersed Fe_3_O_4_ nanoparticles or with gallium, and carbon nanotube wires (CNWs) hybrids are presented. Using these preparation procedures, mixtures of CNTs with different dimensions and chirality, as well as with a variable level of impurities and structural defects, can be produced. The sensors’ performance charts are presented, highlighting a number of aspects regarding the applicability of CNT structures for temperature measurement ranging from cryogenic temperatures to high temperatures, the limitations they have, their characteristics and advantages, as well as the special situations that may arise given the particular structure of these new types of materials, together with basic relationships and parameters for CNTs characterization. Further research will be required to develop the techniques of manipulating and depositing individual CNTs on supports and electrodes for the development of temperature sensors.

## 1. Introduction

Temperature is a representative parameter for a wide range of industrial processes. It is estimated that temperature sensors comprise 70–80% of the global sensor market [1]. Temperature measurement is not performed directly, is based on a series of physical phenomena whose characteristics depend on temperature such as: volume (expansion) and gas pressure variation, variation in magnetic susceptibility, exchanges in the diode junction voltage and electrical resistance, the generation of electromotive voltage (thermocouple), thermal radiation of bodies at high temperatures, etc. Temperature measuring instruments, used in the technical field, can be classified into three categories:

- with direct contact with the media of interest (e.g., a thermocouple immersed in liquid);
- with indirect contact, by applying a temperature-sensitive material on a surface and remotely observing it (e.g., fluorescence thermography);
- without contact, by remote measurement (e.g., infrared thermography).

At the most basic level, a thermometer is a device with a measurable output that changes with temperature in a reproducible manner. 

Precision of temperature measurement is a very critical issue in many scientific and industrial fields, especially in cryogenics. Temperature is the most important parameter when it comes to cryogenic domains. Based on different temperature-dependent properties [2], a variety of cryogenic temperature sensors have been developed. The most widely used temperature measurement equipment in research laboratories, pilot plants, or large cryogenic facilities are: resistors, transistors, diodes, thermocouples, and capacitors. Miniaturization of temperature sensors is still a challenge, especially when the process requires measurements in specific point locations, fast response time, high sensitivity and stability over time, interchangeability, low cost, compatibility with the environment (magnetic, ionizing radiation), simplicity of the system, and low energy consumption that results in a low heat dissipation rate [3]. 

The superior thermal, electrical, and mechanical properties of CNTs [4,5,6] have enabled the development of new types of sensors that use CNTs as a sensing element (temperature, pressure, humidity, gas, and electrochemical biosensors) [7,8,9,10,11] that can be used for many applications (biomedicine, automotive, food industry, environmental monitoring, agriculture, manufacturing industry, security, etc.) [12]. In this paper, we present a short review of different constructive designs of CNTs based resistive sensors used for temperature measurement, available in literature, highlighting the main features and their advantages, the limitations as well as some aspects regarding the applicability of CNTs for temperature measurements at cryogenic temperatures.

## 2. General Considerations Regarding CNTs 

Since their first presentation to the scientific world in 1991 by Iijima [13], at the Japanese NEC Corporation, carbon nanotubes (CNTs) have attracted the attention of specialists from different domains. Research on carbon nanotubes was greatly stimulated by this first scientific paper on the observation of nanoscale carbon tubes [13] and, subsequently, theoretical and simulation works have been conducted to understand this nanoscale material and related phenomena [14]. 

A series of publications followed with observations on the technological conditions required for the synthesis of large quantities of nanotubes [15,16]. The emergence of these early studies led to the intensification of investigation, the results highlighting that carbon nanotube belong to a family of fullerenes structures and can be seen as hollow cylinders, rolled-up graphitic layers into cylinders [17] except at the ends. Carbon nanotubes can be formed by one or more graphite layers, under the form of hexagonal networks of carbon atoms bonded in the sp2 hybridization state [18] except that, in some cases, tube diameters are small enough to present the effects of a one-dimensional (1D) periodicity [19,20].

By discovering the preparation methods of single-wall nanotubes [19,21,22] it is now possible to test and verify predictions and theoretical calculations previously performed. After rolling the graphene sheets, a rearrangement of the marginal carbon atoms to form the nanotube caps takes place. There are cases where the nanotube does not appear closed at the ends. In this case, edge effects are manifested by an increased chemical reactivity of atoms in the bond formation with different radicals.

Depending on the arrangement of the graphene cylinders, there are three types of nanotubes: single-walled nanotubes (SWCNTs), double-walled nanotubes (DWCNTs), and multi-walled nanotubes (MWCNTs). In the case of SWCNTs they have approximately 1 nm diameter and are typically 1–100 microns in length.

**Figure 1:**
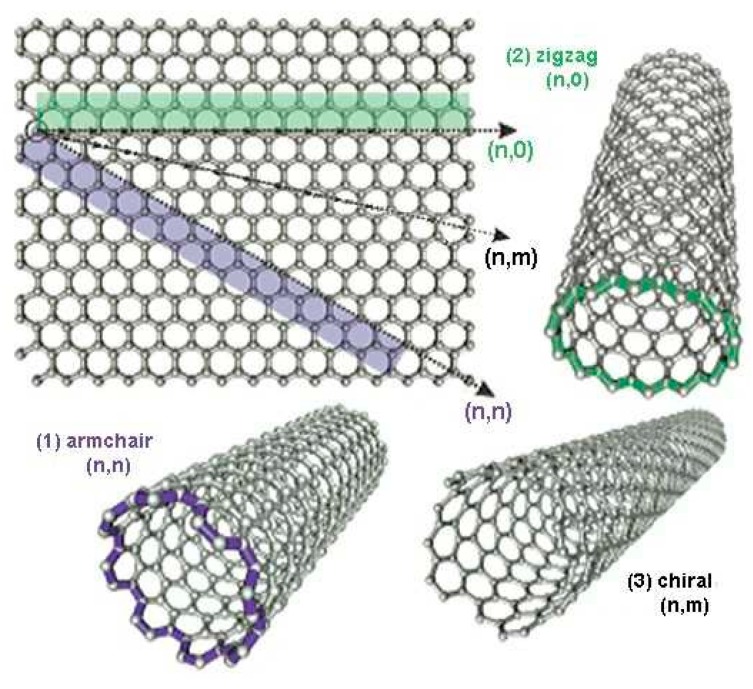
The imaginative process of forming a SWCNT by rolling a graphene sheet in different directions.

In theory, SWCNTs are obtained by twisting graphene sheets having honeycomb-distributed carbon atoms (Figure 1) [14]. Their geometrical structures which are uniquely specified by a pair of chiral indexes (n,m) are directly associated with their electronic properties. Depending on the orientation of the graphene lattice with respect to the tube axis they are twisted, three typical types can be obtained: armchair (n,n), zigzag (n,0), and chiral (n,m). If n-m is divisible by 3, the SWCNT present metallic behavior, otherwise they present semiconductor behavior [23]. 

Of particular importance to the properties of carbon nanotubes are the many possible geometries that can be made on a cylindrical surface without introducing stress factors into the carbon nanotube. For 1D system, on a cylindrical surface, symmetry with a screw shape axis can affect the electronic structure and associated properties. Nearly exotic electronic properties of 1D nanotubes are predominantly resultant of band structure of SWCNTs [24], intra-wall interactions between multiple layers within the same single nanotube (for MWCNTs) [25] rather than between two different nanotubes.

This interesting structure provides them with unique electrical, mechanical, physical, optical, and chemical properties coupled with a high aspect ratio. Those properties are summarized in Table 1 [4,5,6,14,18].

Owing to these superior material properties, CNTs suggest that their use as very sensitive sensing elements in the sensors domain will allow for further designing and development of measurement devices, with superior characteristics to others of similar size. Their high mobility and ballistic transport characteristics, for example, make them serious candidate for the replacement of Si in future devices, especially when miniaturization—as one of the solutions for improved performance—is becoming more and more difficult. Field effect transistor (FET) compatibility and small intrinsic capacitance for possible operation at terahertz frequencies are advantages over Si technologies of similar design [26]. Challenges are related to the reproducibility of properties from one device to another and to their homogeneous growth, without defects, with the desired orientation and the necessary length. 

Their mechanical properties—including high strength, high rigidity, and low density—make them highly attractive for various applications by controlling the band structure and thereby modifying the electronic transport properties. Most importantly, this can be achieved reversibly, opening the way to the vast possibility of designing electromechanical sensors, high current field effect transistors, and low resistance interconnects in electronic devices [27].

However, there is currently no transducer available on the market, that incorporates a sensing device capable of exploiting the superior properties of carbon nanotubes due to the fact that nanoscale manufacturing processes are still very costly and technologically advanced. 

Although many companies have activities in place related to the integration of carbon nanotubes in their currently manufactured products, the products offered on the market are still in their young phase of the life cycle. To be at the cutting edge of the nanotechnologies, due to the globalization of the market, it is a recipe for future achievements and for preservation of market share. With the notable domains of coatings, composites, and energy, there are only few companies from microelectronics selling CNT-based products [6]. There is a strong market expectation from the domains of transparent conductors, thermal interfaces, wind turbine blades, and antiballistic equipment to be areas of excellence following the implementation of carbon nanotubes. 

Historically, in the field of electronics components and electronics manufacturing, the technological advance caused by penetration into the micro-scale constituted a significant development step. It was obvious that smaller electronic devices would mean using less space, better portability, and most importantly, saving materials by using as little as possible. On the other hand, the materials used must be of particular purity, since even in small numbers the impurities can represent a significant percentage of the amount of the used material. 

As technology has advanced, the possibility of building small elements has grown, and through the intensive use of carbon nanotubes, science enters the nanometer scale era. 

In addition to the dimensional differences between the two size scales—micro and nano—there are other considerations that need to be considered for the choice of manufacturing processes. Physical handling is still possible in these dimensions even if it requires precision that is difficult to achieve even with the help of very expensive equipment, such as a powerful microscope. However, in particular, it is even more complicated to physically move, assemble, or modify objects at a nanoscale using standard microfabrication equipment.

For the design and development of nanometer-scale devices, it is essential to discuss and understand what steps are required to successfully manufacture and characterize a device at such a size. One of the most important differences between how ordinary objects (millimeter) and nano-scale objects behave is determined by the forces that control the state of matter. On a regular scale (meso-scale) the dominant force is gravity together with the friction force. In the micro-scale field, dominant forces are surface forces. These surface forces include static friction, friction, electrostatic forces, and van der Waals forces. At the nanometric scale, the main forces are intermolecular and atomic forces, the effects of which are often neglected in meso-scale analyzes. It is therefore important that further research be directed to understanding the hypotheses that can be used to accurately determine the behavior of ultra-small devices. 

Conventional assembly (meso-scale) processes are very simple and involve either the use of the operator’s hand or automated manufacturing equipment. By contrast, micro-scale manufacturing processes require the use of very precise equipment, equipment that cannot be used in the nano-scale field. At the nano-scale, the product manufacturing process requires more complicated steps, including self-assembly and processes that involve direct handling of materials for example. 

At present, cost is an important issue in the development of nanotechnology. ‘Nanomanufacturing’ processes involving individual CNTs, as defined and used by U.S. National Nanotechnology Initiative (NNI) [28], still produce a very small number of products that are sent to the market due to both the lack of specific manufacturing methods, the precision measurement solutions of the physical dimensions and also due to the lack of necessary commercial infrastructure. For example, carbon nanotubes are produced/grown by using multiple technologies, but the final products where they are employed are not promoted on the market in a consistent manner. Most products using CNTs today incorporate CNT powders dispersed in polymer matrices [6]. The reasons are also given by the synthesis processes, which are costly and slow, a fact which currently represents a limitation in the development of nanotechnologies. Even if manufacturing prices are expected to become competitive, it is still too early to discuss the marketing of CNT-based nanomaterial technologies.

## 3. Constructive Solutions for Temperature Sensors Based on CNTs

In recent decades, sensor development has been a topic of interest for researchers and the use of nanomaterials has led to new types of nanosized sensors. Sensor miniaturization has arisen from the need for smaller and faster devices with high sensitivity, low power consumption, fast response time, and ability to installed at precise locations in hard-to-reach areas or where temperature fluctuates rapidly without disturbing the neighboring environment (as the case of a cryogenic system). Because of their good electrical response to the temperature variations, CNTs became a serious candidate as an emerging material to provide solutions for the future temperature sensors development. Both single and multiwall CNTs, having either metallic and semiconductor behavior, have been reported as temperature sensors [11,29,30,31,32,33,34,35,36,37,38,39,40,41,42,43,44,45,46,47,48,49,50,51,52,53,54,55]. 

Besides issues on substrate type, electrical contacts, packing and acquisition method, an important aspect for designing and construction of a temperature sensor based on CNTs is related to integration of the CNTs into the sensor’s structure. The CNTs can be integrated individually, under the form of networks or bundles of tangled CNTs (CNT ‘ropes’) that are electrically contacted between them and with electrodes of different geometries. Another class of CNTs based temperature sensors is centred on the usage of CNTs as reinforcements for different polymer nanocomposites. Those materials, obtained by the integration of CNTs into polymeric matrices, are the structures of choice not only for temperature measurements but also for a large number of potential applications, such as photovoltaic devices [56] aerospace or other industries, where new materials characterized by unprecedented properties are continuously needed. Indicative of an emerging technology, there are only a few nanotube-based commercial products in the market at present [57]. It is expected that the replacement of carbon black, the most common industrial filler material, with CNTs for the preparation of electrically conducting polymer composites is expected to have a great impact on a wide range of industrial applications [58].

The obstacles in making a CNT-based sensors are related to nanostructures integration and nano-manufacturing. There are a few structure designs, fabricated in batch assembly processes [59], but most of CNT-based sensors are produced in a serial assembly process, resulting in a low number of products. Over time, several assembling methods have been developed, including:

- direct growth on the substrate by chemical vapor deposition (CVD) process [29,30,31,32,33,34,53,54,55];
- thin films obtained by gluing [37,38], printing [35,36], filtration over a membrane [51,52], spraying [49,50,60];
- drop-casting deposition from a solution followed by a dielectrophoresis (DEP) procedure [39,40,41,46,47,61,62].

Table 2 emphasis a comparison between different types of temperature sensors based CNTs available in literature. Also, special types of CNTs temperature sensors are presented, including hybrid nanoparticle sensors [63], gallium-CNT sensors [64], and optically-induced dielectrophoresis (ODEP) [65] assembled CNT sensors. The following sections will overview these types briefly.

### 3.1. Characteristics of a Temperature Sensor Assembled by Direct Growing of CNTs on the Substrate

In this subchapter is described the method of direct growth of CNTs on a substrate, between the electrodes, by the chemical vapor deposition (CVD) process, in order to be integrated in the microstructures of a temperature sensor. Examples of CVD processes suitable for CNTs growth include, but not limited to: thermal CVD, plasma-enhanced CVD, vapor phase growth, and laser assisted CVD [11].

The direct growth of nanotubes on the support platform of the sensor by means of CVD methods involves the heating of the catalytic metal nanoparticles at high temperatures (500–1000 °C) in a furnace, then the feeding of the furnace with gaseous hydrocarbons (ethylene, acetylene, or methane) for a certain amount of time [66]. Thus, CNTs are synthesized by transmitting the energy to hydrocarbons; the energy sources used are: electrical resistors, electron beam [12], plasma-enhanced [30], or microwaves [54]. The transmitted energy breaks the hydrocarbons molecule into reactive radical species, then they diffuse to the coated catalyst substrate (Ni, Fe, Co, or metal alloy) [12]. The most commonly used substrate is Si, Si coated with a Si dioxide insulating layer, but also glass [32], aluminum oxide [67], ceramic or Sitall (crystalline glass-ceramic with ultra-low coefficient of thermal expansion) [40] are used.

A first step in the manufacturing of a microsensor is to fabricate the electrodes on the support platform in order to obtain an ohmic contact and very good electrical connections. There are different methods and materials used, presented in the literature. The most common presented method is the photolithography of metallic contacts (Ti, Pt, Au, Ag) [53,54,68] of a certain form, such as golden islands on the ends of a nanotube or over the entire network of nanotubes. De Volder [29] deposited, by sputtering, a layer of 100 nm TiN thick on a silicon plate. Then, to obtain the electrodes, the desired pattern in the TiN layer is outlined by the template removal method. A less common method is to obtain electrodes by gluing Ag slurry at the ends of the nanotube network, already grown on the support [32].

The next step, before applying the CVD process for nanotube growth, is the deposition of the catalyst. Typically, the catalyst is deposited in a thin layer, in the order of nm, on metal electrodes by the optical lithography method [29]. Other catalyst deposition techniques such as ion beam deposition [32], sputtering [54,55], or ink-jet printing [69] on the substrate can be encountered. However, Pal [70] and Friedman [47] growth MWCNTs by a catalyst-free CVD process, within the pores of alumina templates.

In the frame of CNTs synthesis method by CVD technique, some parameters have to be taken into account to achieve the desired results, the most important being: the nature of the hydrocarbon, the catalyst and the temperature of growth. The most common CVD techniques use methane, ethylene, acetylene, and carbon monoxide [55] to grow CNT of multi-wall type (MWCNT). Chaisitsak [71] observed that optimizing growth conditions (catalyst and temperature) obtained both single-wall and multi-wall CNTs. Also, the density and growth rate of CNT increased as temperature rose, and there was observed a tendency for vertical alignment. 

In all these cases, the manufacturing methods are complex and cumbersome and the yield is low. To ensure the success of sensors of any kind that rely on CNTs, we need cheap and good manufacturing technologies that can be reproducible [72]. A high quality sensor can be obtained through strict control of CNT growth conditions. Uniform growth of the CNTs is a challenging job as they are sensitive to many factors (surface roughness, flow of source gas, temperature, etc.) [55]. So, *Sarma* [55] obtained a uniform MWCNTs thin film temperature sensor by optimizing the growth process parameters: deposition time, inside temperature and process gas to carbon source gas ratio. The sensor was tested it in the temperature range 22–200 °C, presenting a temperature coefficient of resistance (TCR) of 1.03 × 10^−3^/°C and a sensitivity of 3.3 × 10^−3^ V/°C.

**Figure 2:**
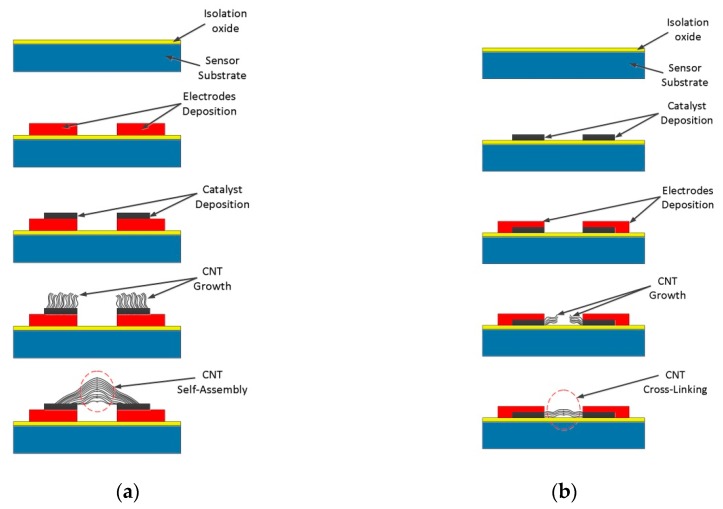
Fabrication process of the CNT based sensor using CVD technique: (**a**) vertical growing and self-assembling; (**b**) horizontally growing. Reproduced from [29], courtesy of Michael De Volder.

Individual nanotubes or vertically aligned nanotubes were grown directly on the electrodes. However, nanotubes are difficult to integrate into a temperature sensor because there is no electrical contact at both extremes, thus resulting in low temperature sensitivity. This problem has been solved by researchers by developing a capillary self-assembling method [29,54,73,74,75,76,77,78], where MWCNTs are grown directly on the electrodes, in vertical or horizontal plane, forming a bridge and finally resulting an interconnection between electrodes (Figure 2). Tawfick [79] realized a horizontally aligned CNT film on the Si substrate by mechanical rolling of vertically grown MWCNTs. 

**Figure 3:**
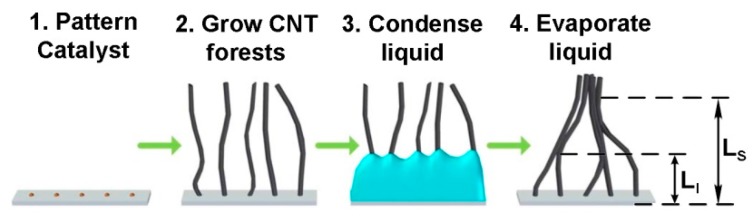
Successive steps used in the CNT capillary self-assembly process. Reproduced from [29], courtesy of Michael De Volder.

Verploegen [74] described the capillary self-assembling of MWCNT microstructures in the vertical plane as a means of increasing the density of nanotubes directly on the electrodes and to connect them, using elasto-capillary densification with an organic solvent. Figure 3 illustrates the successive steps used in capillary self-assembly of nanotubes [29,74]. 

After the catalyst is applied to a support and the nanotubes are grown by the CVD process, a solvent is applied and condensed onto the support, for example ethanol. Due to capillary growth, the solvent is withdrawn into each CNT structure by increasing its density. During solvent evaporation at ambient conditions, each CNT structure is individually modeled by forces resulting from capillary action [77]. The CNTs are joined together, two by two, at a certain distance *L_I_* from the substrate (Figure 3). Furthermore, at a distance *L_S_*, a pair of CNTs will aggregate with another pair, forming a bundle and this process continues until micro-sized bundles are formed. Py [80] described a model to where *L_I_* and *L_S_* are determined by

(1) $$L_{I}={\left[\frac{9}{2\left(\pi -2\right)}\right]}^{1/4}\sqrt{d\sqrt{\frac{E\pi R^{3}}{4\gamma }}},$$

(2) $$L_{S}=L_{I}{\left[\frac{\beta^{2}\left(\pi -2\right)}{2\sqrt{\pi }3^{1/4}\left(2-\sqrt{2}\right)}\right]}^{1/4}N^{3/8},$$

where *d* is the spacing between adjacent CNTs, *E* is the Young’s modulus, *R* is the CNT radius, *γ* is the surface tension (0.025 N/m for ethanol), β accounts for the lattice geometry, and *N* is the number of individual CNTs in the bundle. The above equations describing elastocapillary aggregation are useful in the process of assessing the density of the catalyst deposition on support, subsequently the measurement of the density of the grown nanotubes, the height of the formed nanotubes forest, the diameter of the obtained bundles, and their distance from the substrate [74].

**Figure 4:**
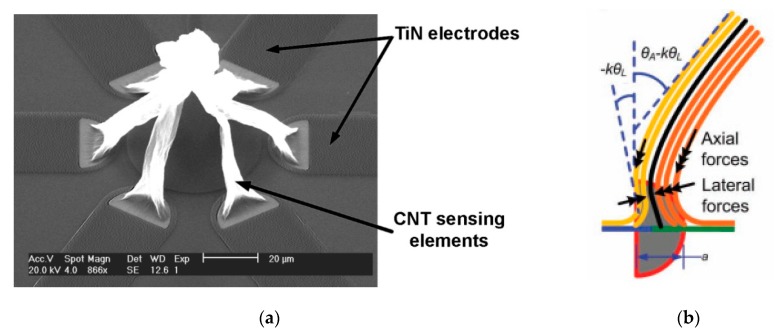
(**a**) SEM images of the CNT based temperature sensor investigated by *de Volder*. Reproduced from [29], courtesy of Michael De Volder; (**b**) forces applying on CNTs during self-assembly process from a semicircular area [77] Copyright © 2010 WILEY-VCH Verlag GmbH & Co. KGaA, Weinheim.

**Figure 5:**
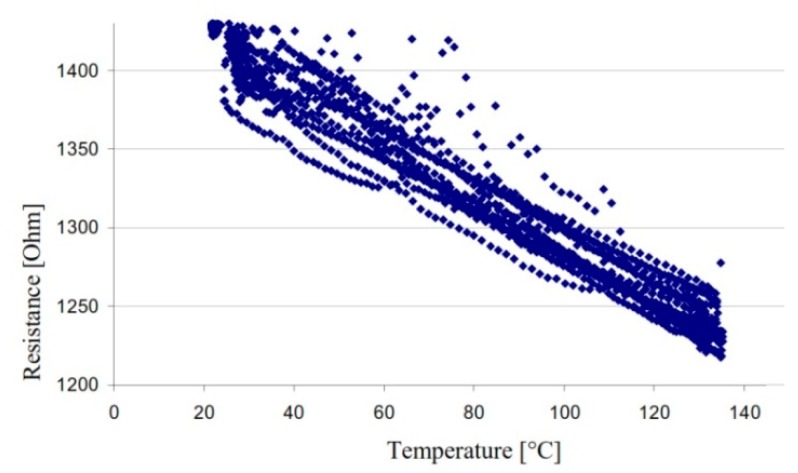
Temperature–resistance curve. © 2010 IEEE. Reprinted, with permission, from [29].

The final shape of the CNT structure thus modeled is maintained by the intermolecular attraction forces and by the mechanical interlocking due to the alignment state of the CNT. Depending on the initial geometry of the catalyst on the substrate, new 3D geometries can be created from CNTs. Figure 4a shows a temperature sensor developed by de Volder [29] through the self-assembly process in a 3D bridge model. This has resulted in MWCNT networks suspended over a substrate, which will allow for a faster response time than in the case of sensors created from a thin film in direct contact with the substrate. The investigated geometry consists of six TiN electrodes arranged circularly, each electrode ending in a semicircular area for MWCNTs growth. A schematic of lateral and axial forces during capillary forming of a semicylindrical forest is presented in Figure 4b [77]. Following the self-assembly process, a 100 μm diameter bridge made of six MWCNTs branches is the result. The sensor thus obtained was tested in an enclosure having a constant temperature in the range of 20–130 °C. The temperature-resistivity characteristic of the nanotube bridge (Figure 5) showed a TCR coefficient better than −0.1 %/K over numerous measuring cycles.

Another way to grow and interconnect CNTs by CVD method is in the horizontal plane between the sensor electrodes. To stop the growth of nanotubes in the vertical plane, Han [73] deposited silicon oxide over the Ni catalyst so that only the lateral side remains exposed to the growth of the nanotubes, in a horizontal plane as in Figure 2b. An important aspect to be taken into account when applying this method is the distance between CNT growth electrodes. An excessive distance leads to the impossibility of interconnecting the electrodes.

**Figure 6:**
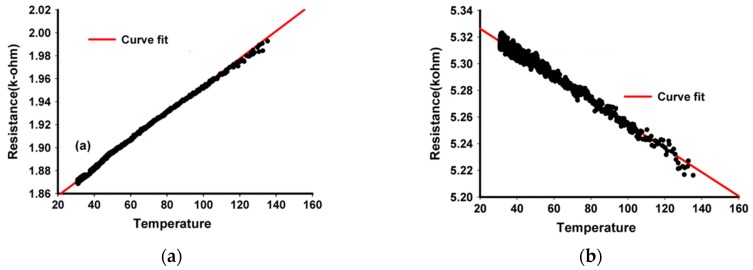
Sensor temperature–resistance curves for MWCNTs grown with (**a**) 0.9 μm and (**b**) 1.8 μm gap between electrodes. © 2007 IEEE. Reprinted, with permission, from [54].

Kuo [54] has built structures, with different sizes between electrodes between 0.6 and 2.7 μm, in an ECR CVD (electron cyclotron resonance chemical vapor deposition) deposition enclosure, microwaves powered, MWCNTs were grown, having looped form. The used gas source was a mixture of CH4 with N2, with the CH4 concentration varying between 10% and 60% respectively, and the gas flow between 20 and 60 sccm. The enclosure pressure was 30 torr and the substrate temperature was 400 °C. It has been observed that in order to obtain high quality MWCNTs, the CH4 concentration must be 40%. At a concentration of less than 40%, the possibility of carbon atoms reaching the active growth center of the catalyst is lower due to the low exposure angle (90°). The higher the methane concentration is over 40%, the more the nanotubes become loose and with impurities, resulting in fewer CNTs connecting the electrodes. At higher flows, due to the etching phenomenon of nitrogen gas, both a decrease in the number of the obtained CNTs and their length was observed. Also, the number of CNTs connecting the electrodes decreases as the distance between them increases, resulting in a decrease in conductivity. Finally, the sensors thus obtained were tested as temperature sensors in the range 30–140 °C, obtaining a linear variation of resistance with temperature and a TCR between 0.0008152 and 0.0001759. Figure 6 shows the results obtained for two sensors having the distance between the electrodes of 0.9 μm and 1.8 μm, respectively, under the same conditions of MWCNT growth. It is noticed that two types of MWCNTs, with metal characteristic (Figure 6a) and semiconductor characteristic (Figure 6b) were obtained due to changes in the structure of the carbon network. There are rare cases in which semiconductor MWCNT sensors are obtained.

**Figure 7:**
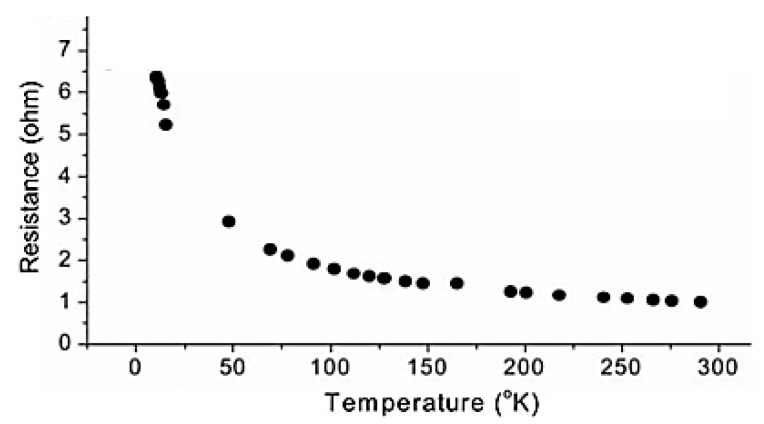
Resistance–temperature curve of a cryogenic sensor based on CNTs vertically grown by CVD method [32], reprinted by permission of the publisher (Taylor & Francis Ltd).

A cryogenic temperature sensor, having as sensitive element CNTs grown using CVD, was performed and tested in the range 10–300 K (Figure 7) [32]. It is noted that the sensor has a semiconductor characteristic, with a drastic change in resistance at cryogenic temperatures of more than 600%. 

### 3.2. Characteristics of Temperature Sensors Based on CNTs Thin Films Assembles by Gluing, Printing, Filtration over a Membrane, and Spraying

For the integration of CNTs thin films, as sensitive material, into microstructures, in order to obtain temperature sensors, several methods have been employed. In this subsection, we briefly present the characteristics of the following assembling procedures: gluing, printing, filtration over a membrane, and spraying.

**Figure 8:**
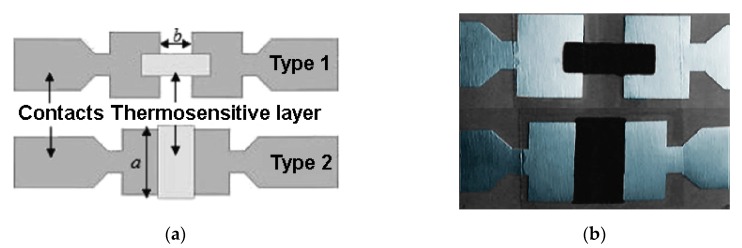
Schematic (**a**) and optical image (**b**) of the sensors structure obtained by printing [35].

Recently, MWCNTs have been successfully integrated into flexible electronic devices for temperature measurement [35,36,37,38]. Flexible temperature sensors have become attractive to researchers because of their ability to improve the functionality of integrated bio-parameter monitoring systems such as the body temperature control systems of a soldier in the battlefield or of a patient. Karimov [37,38] obtained flexible temperature sensor by deposition of MWCNTs powder between adhesive elastic polymer tape [37] and on paper substrate [38]. To better adhere the film to the paper substrate, a pressing procedure was applied. The sensors terminals were connected to the sensitive film by silver paste and tested at temperature range 20–70 °C, presenting semiconductor behavior with a maximum TCR of −1.26 %/°C for the MWCNTs film on the elastic polymer tape substrate. Walczak [35] ave studied a temperature sensor based on a thermo-sensitive layer made from MWCNTs deposited on a flexible polyimide support using the screen-printing method, a cheap and scalable method that is extensively used on an industrial scale. The composition that was printed contained poly (methyl methacrylate), an organic solvent, and MWCNTs mixed with an organic resin as filler [81]. To prevent agglomeration of the nanotubes in the bundles and disperse them evenly into the solution, the composition was ultrasonicated in an ultrasound bath before printing. The used flexible substrate was made of KAPTON® HN, a high temperature polyimide, on which gold-plated copper electrodes were originally made using anodizing method. The golden layer was deposited in order to reduce the contact resistance and to prevent oxidation. After printing the thermo-sensitive layer based on CNT, the sensor was subjected to a thermal treatment to give it flexibility and stability. Finally, the sensor was encapsulated with adhesive tape to protect it mechanically and against the moisture. By using the method described above, Walczak [35] made four sensors with different concentrations of CNTs (0.25 wt % and 1 wt %) and different sizes of sensitive layers (Figure 8), which were tested in the temperature range 33–43 °C.

**Figure 9:**
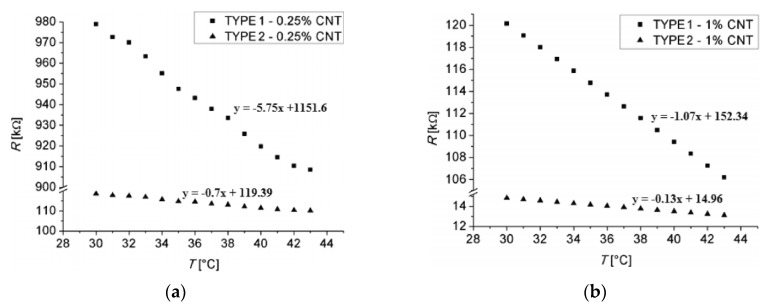
Temperature–resistance curve of sensors with (**a**) 0.25 wt % CNT and (**b**) 1 wt % CNT [35].

A linear negative characteristic of resistance variation with temperature was observed in all situations (Figure 9), with a TCR of approx. 2-times higher in the case of sensors with 1 wt % CNTs, without any noticeable influence after the bending of the sensitive element.

**Figure 10:**
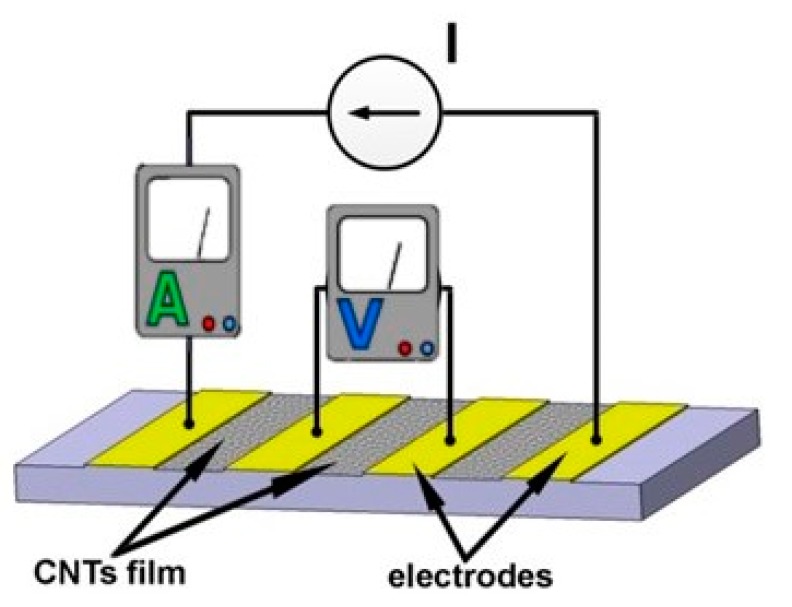
Measurement setup for a temperature sensor based on freestanding CNTs thin film.

Thin films were also obtained by vacuum filtration over a support membrane of a solution containing CNTs [51,52]. Thus, 0.5 g of high purity MWCNT (> 97%) was uniformly dispersed by sonication in 100 g of water containing 0.1 mg of sodium dodecyl sulfate. This solution is filtered by vacuuming through a membrane, resulting in a thin film of MWCNT (freestanding), stable, not supported by another structure, or by applying a chemical agent to bind CNTs. The thickness and density of this film can be controlled by the amount of filtered solution, and then, after drying, it can be cut to the desired shape on which metal electrodes can be built. Thus, di Bartolomeo [51] has obtained a sensor 3 x 6 mm in size and with a thickness of approx. 300 μm, on the surface of which four electrodes were drawn from Ag with a distance of 1–2 mm between them (Figure 10).

**Figure 11:**
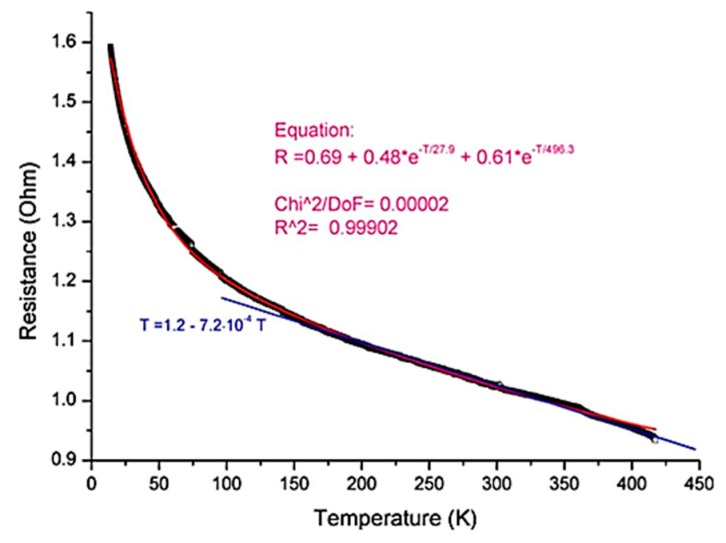
Resistance–temperature curve of the cryogenic sensor obtained by *di Bartolomeo* [51]. Copyright © 2009 American Institute of Physics.

Next, several thermal cycles were performed on the sensor from room temperature to 100 °C in order to stabilize the strength of the CNT film. By using the temper procedure, di Bartolomeo [51] assumed that the contact between the CNTs and between the CNTs and the Ag electrodes is improved, and also the impurities and adsorbents are evaporated. Finally, di Bartolomeo [51] tested the CNT sensor at low temperatures down to 4.2 K, observing a rapid and monotonous response of resistance variation with temperature, with a TCR of −7 × 10^−4^ K^−1^ in the range of 150–420 K (Figure 11).

The spraying deposition technology is a high-performance and cost-effective technology used to produce high-quality films (uniform density of CNTs), over almost any sensor structure, at room temperature. An important aspect to be taken into account when applying this method is the necessity of uniform dispersion of CNTs in the solution (like an ink) before to be applied to the sensor support. This uniform dispersion is necessary because, due to the van der Waals forces existing between them, the CNTs have a tendency to aggregate. To avoid this inconvenience, a sonication procedure shall be applied. 

**Figure 12:**
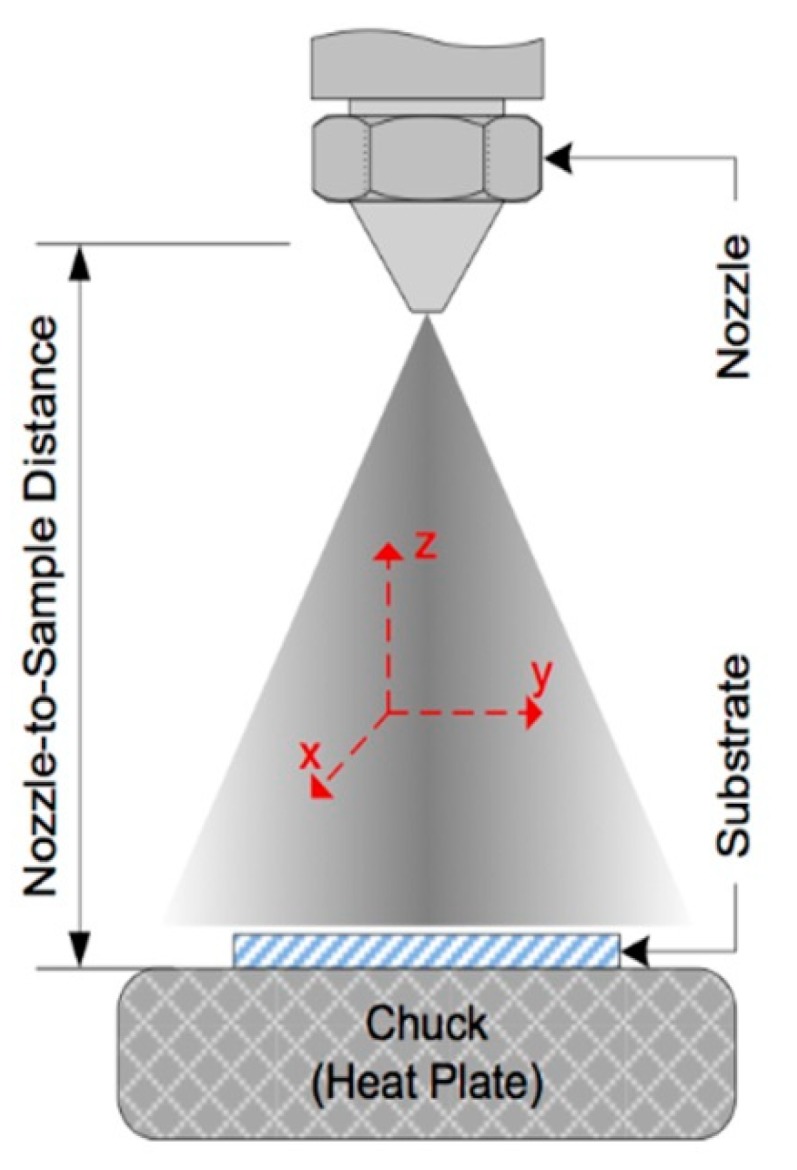
Schematic representation of the spraying device used by Cagatay. © 2014 IEEE. Reprinted, with permission, from [50].

For the deposition device, the spraying nozzle, Cagatay [50] used an automatic system consisting of an air atomizing spray valve and a mobile platform, installed in the upper part, where the controlled parameters were: the flow rate of the solution, spraying gas pressure, distance between the orifice and the sensor support, temperature of the substrate, and speed of movement of the platform (Figure 12). 

The CNTs have been deposited in a multi-layered mode, layer-by-layer, ultimately obtaining a high density CNTs film. The sensor was successfully tested at temperature variation in the 0–80 °C range, with a TCR coefficient of −0.002954 K^−1^.

### 3.3. Characteristics of a Temperature Sensor Assembled Using Drop-Cast/Dielectrophoresis Method

**Figure 13:**
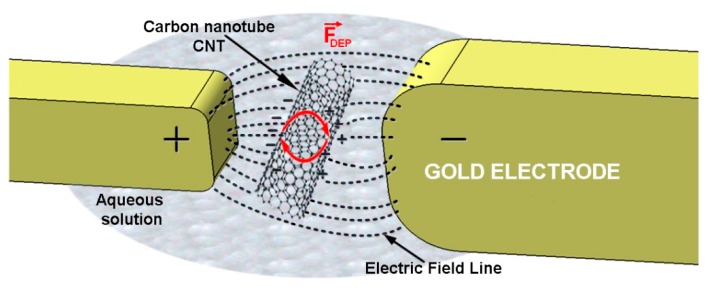
CNT undergoing DEP motion.

The advances made in nanotube chemistry research have enabled both the dissolution and dispersion of nanotubes in different solvents [82]. This fact provides new alternative routes for the manufacturing of nanotube models by simply dispersing or printing particles dissolved or dispersed on the substrate, which results in the obtaining of randomly arranged nanotubes on the support substrate. The electrical resistance of the sensor, depending on the density of the nanotubes between the contact electrodes, can be adjusted in a simple way by adjusting the concentration of nanotubes dispersed in the solution or by adjusting the volume of solution used. Usually, after the deposition of the CNTs solution by a drop-cast method with a nano or micro syringe, a dielectrophoresis (DEP) process is required in order to align the nanotubes along the electrodes [39,41,61,62]. DEP is a phenomenon where neutral particles undergo mechanical movement within a non-uniform AC field (Figure 13) [39]. After that, an annealing process is necessary for solution evaporation, CNTs better adhesion on support [40]. Friedman [47] hydrogenated MWCNT by annealing them in Ar/H_2_ atmosphere at 800 °C, which induces ferromagnetism. 

The DEP force direction depends on the electrical properties of both nanomaterials (CNT) and the suspension medium (insulating dielectric fluid, for example ethanol or dimethyl formamide—DMF). In the case of an AC current the DEP force is determined with the relations [83]

(3) $$F_{DEP}\left(t\right)=2\pi ab^{2}\varepsilon_{m}Re\left(K\right)\nabla {\left|E_{rms}\right|}^{2},$$

(4) $$K=\frac{\epsilon_{p}-\epsilon_{m}}{3\left[\epsilon_{m}+\left(\epsilon_{p}-\epsilon_{m}\right)L\right]},$$

where ∇|E_rms_ |^2^ is the gradient of the root mean square of the electric field, *a* and *b* are half the length of the CNTs and the radius of the CNTs, respectively, $\epsilon_{m}$ and $\epsilon_{p}$ are the permittivity of the environment and of the CNTs, *L* is the depolarization factor (L≈ (b^2^⁄a^2^)[ln(2a/b)−1)]), *K* is the complex polarizability factor (Clausius–Mosotti factor) that shows the interrelationship between the frequency dependent properties of the CNTs and the environment. When Re(K) is greater than 0, the assembly process is performed by a DEP positive force (PDEP), otherwise it is performed with a negative DEP force (NDEP).

For PDEP assembling, CNTs are attracted to regions where the electric field strength is highest, instead, for NDEP assemblies CNTs are attracted to regions where the electric field strength is the lowest. For example, in order to incorporate SWCNTs on 3D electrodes [84] a 10 MHz frequency and PDEP force can be used [85]. By manipulating the parameters described in (3) and (4), such as voltage and frequency, and taking into consideration that the distances between electrodes and dielectric properties are known parameters, the overall position of nanotubes can be estimated.

Following the DEP process, a process of hardening (by heating) or blowing the structure can be applied to remove the remaining CNT solution and to improve the adhesion to the support of the CNT film aligned between the electrodes.

**Figure 14:**
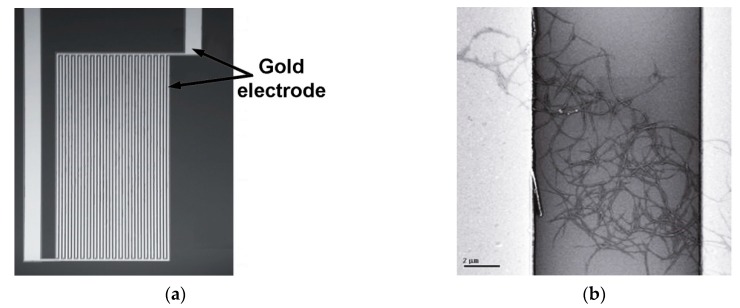
(**a**) Interdigitated gold electrodes on a Si substrate; (**b**) SWCNTs deposited between electrodes. Reprinted with permission from [86]. Copyright © 2003, American Chemical Society.

The most popular support platform for CNT-based sensors, presented in literature, is a Si substrate, on which interdigitated electrodes from Au, Pt, or other high-conductivity metals are photolithographically printed. Such a platform is presented in Figure 14, on which SWCNTs have been deposited and aligned.

**Figure 15:**
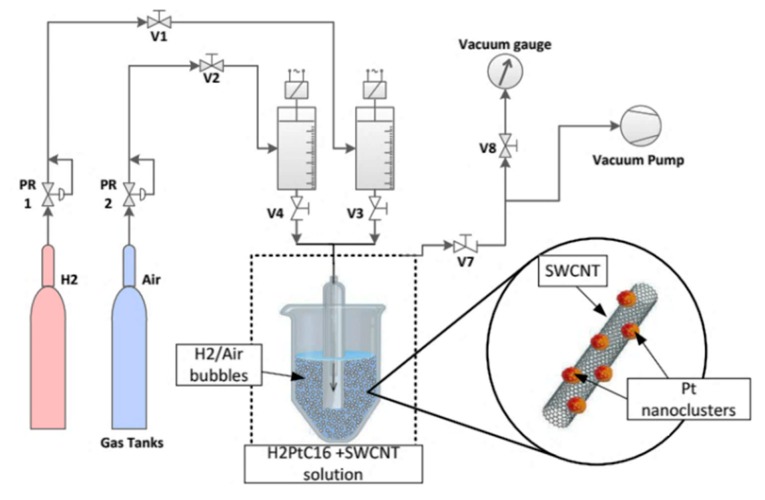
Schematic view of bubling experimental set-up for CNT decoration with Pt nanoclusters [40].

A SWCNT cryogenic temperature sensor assembled by the drop-cast and DEP procedures was investigated by Monea [40]. Commercially produced SWCNTs (purchased from Sigma-Aldrich, 60% purity) were mixed together with isopropyl alcohol (1/10 weight ratio). The resulting solution was homogenized in an ultrasonic bath, then filtered and dried. A deposition process of Pt nanoparticles on CNT was carried out by bubbling of high-purity H2 over the SWCNT solution mixed with 1 wt % chloroplatinic acid hexahydrate (H_2_PtCl_6_) in distilled water (Figure 15). The role of Pt nanoparticles is to facilitate the adhesion of SWCNTs between electrodes and to improve the conductivity of the structure.

**Figure 16:**
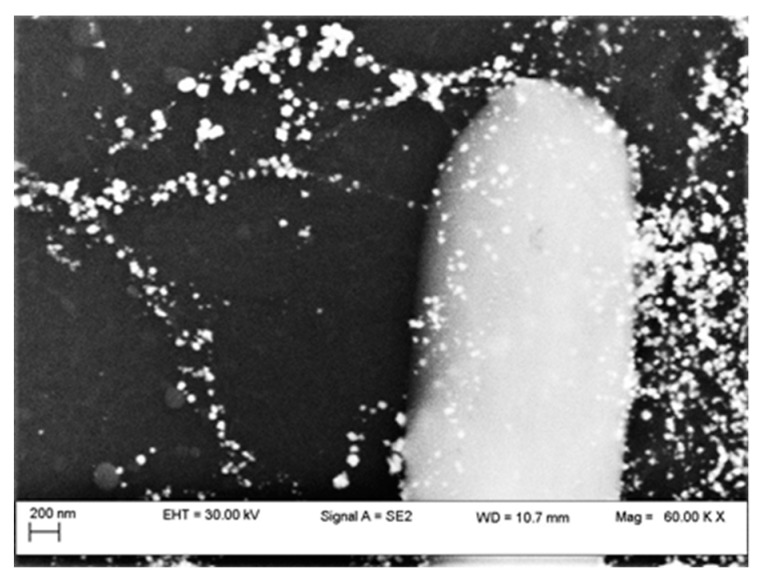
SEM image of a part of the sensor structure [40].

A quantity of 10 μL of SWCNTs solution was dropped between interdigitated gold electrodes on a Sitall substrate (a mixture of crystalline glass and ultra-low thermal expansion ceramic). For the alignment of the CNTs between the electrodes, a potential of 1 VDC was applied to the electrodes, and dried air heated at 60 °C was blown. In order to obtain a stable active layer, the structures were conditioned at 200 °C for 1 hour. Figure 16 shows the SEM image of a part of the structure in which aligned SWCNTs, with Pt deposited on the surfaces can be observed. The sensor thus obtained was tested at temperature variations in the range of 1.9–300 K.

**Figure 17:**
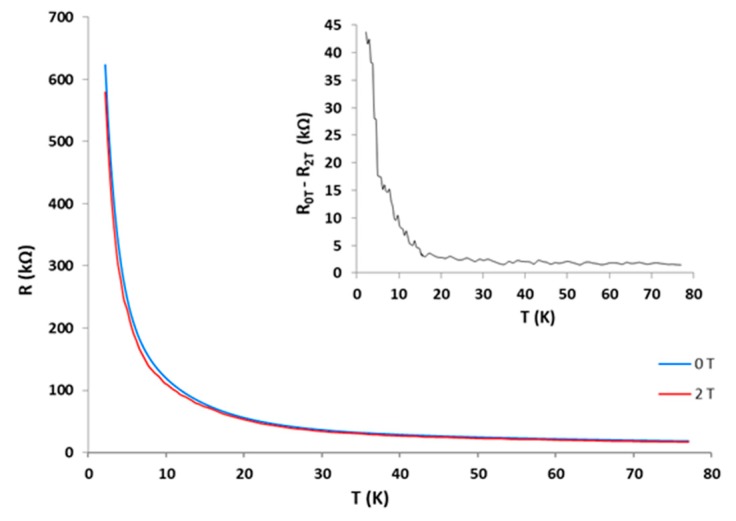
Resistance–temperature curves for sensors at a magnetic field B = 0 T and 2 T. The inset shows the difference of the resistance measured at B = 0 T and 2 T [40].

The sensor thus obtained was subjected to repeated cooling cycles from room temperature to approx. 2 K, a high sensitivity and rapid response to temperature variation was observed, especially at temperatures below 20 K, with a variation of TCR coefficient of −1.473 %/K (Figure 17). Additionally, the influence of the magnetic field on the sensor in the temperature range 2–77 K was studied. The inset of the Figure 17 it is presented the temperature dependence of the resistance under the influence of a magnetic field of B = 2 T (Tesla), compared with the characteristic measured at zero field [40]. The conclusion was that the sensor is sensitive to the magnetic field with a steeper drop in resistance, especially at temperatures below 15 K.

### 3.4. CNTs Nanocomposite Structures Used for Temperature Measurement

Polymer nanocomposites reinforced with carbon nanotubes have generated interest in the field of sensors in recent years. Although for temperature sensors the tests were performed approximately in the range 0–100 °C, far away from cryogenic temperatures domain, from different reasons such as the creep behavior, the stress generated by contraction–relaxation aspects and the compliance of the polymeric material with temperature, to name just a few, since the efficiency of this type of sensors have been proved in other domains it is necessary to make a brief presentation.

At present, the most challenging difficulty is to obtain a uniform dispersion at the nanoscale level, this being an absolute requirement for the complete translation of nanotubes interesting properties to the composite resulting material. Solution blending, under the form of direct mixing of the nanotubes with polymer, is the most used and effective method for preparing nanocomposites [87]. The procedure involves the preparation of nanotube dispersion in a suitable solvent, followed by the mixing with a polymer or a polymeric solution and the preparation of the resulting sensitive film [42]. Although numerous efforts have been made, due to the non-reactive nature of CNTs surface, successful dispersion remains a challenge. Several CNT nanocomposite structures are presented along with attempts to develop numerical methods to predict electrical resistivity according to temperature [11,38,42,44,45,88,89,90,91,92,93].

The fabrication of a temperature sensor based on a MWCNT/ styrene-b(ethylene-co-butylene)-b-styrene (SEBS) triblock copolymer composite is reported by Matzeu [42], with the sensor supports made of polyimide film Kapton, thickness 50 μm. The devices were investigate in the 20–60 °C range, having a sensitivity of −20 ± 5 Ω/°C, for a newly made sensor, which became −3 ± 0.4 Ω/°C after use. Using thermo gravimetric analyses it has been demonstrated that the employed procedure for the preparation of the dispersion was reproducible, with a coefficient of about 2%, also the films sensing properties were reasonably reproducible. Three sensors were prepared from the same dispersion, and investigated under the same conditions, the maximum deviation of electrical resistance from the average resistance value, through the whole range of temperatures, was determined to be 8%.

Ounaies [88] reported electrical properties of SWCNTs reinforced polyimide composite as a function of SWCNT concentration. An aromatic colorless polyimide CP2 was selected as a polymer matrix and, using a method developed by the authors, a series of SWCNTs-CP2 composites were prepared by in situ polymerization under sonication. The resulting degree of SWCNT dispersion was very high, the obtained films having a SWCNT concentration ranging from 0.01 to 1 vol %. The resulting structures, having 2.54 mm in diameter and thickness from 20 to 40 μm, were DC evaluated as being a resistor and capacitor in a parallel connection. A sharp increase of the conductivity value from 3 × 10^−17^ to 1.6 × 10^−8^ S/cm has been determined when SWCNT concentration increases from 0.02 to 0.1 vol %, the pristine CP2 polyimide conductivity being around 6.3 × 10^−18^ S/cm. At loading levels in excess of 0.1 vol.%, the conductivity increased only moderately, being 3 × 10^−7^ S/cm, 10 orders of magnitude higher than the value at 0.02 vol %, strong indication of percolation threshold, in other words the presence of transition between electrical insulator and conductor.

The fabrication and temperature sensing properties of the CNTs-Silicon nanocomposites based sensors are presented by Chani [44] and Karimov [46]. The silicon (Si) powder was obtained by the milling of p-Si crystal wafer and as adhesive material the Hero Gum (Si adhesive) and GMSA (organic polymer) were used. Four different types of samples were prepared by the sequential use of drop casting and doctor blade technology on glass substrate. Resistance temperature relationships of the sensors were investigated in the range 20–90 °C, where a decrease in the resistance of 12–29% was recorded. The initial resistance value of the obtained structures was in the range of 263 to 34 kΩ depending of composition, ratio of components and kind of adhesive material. It was found that the sensitivity of the sensors was in the range −0.53 %/°C to −0.74 %/°C (Table 2), comparable with the sensitivity of platinum based resistance sensors.

An epoxy layer, mixed with a small concentration of MWCNTs was used as an ultra-low cost temperature sensor by Neitzert [89]. The composites were obtained by mixing 0.5 wt % MWCNTs sonically dispersed for 20 min into diglycidyl ether of bisphenol A-epoxy resin (DGEBA) with 4.4”diamine-dibenzyl-sulfone hardener (DDS). The resulting composite was molded into a cavity of 100 × 30 × 2 mm where, after hardening process two electrodes were attached by application and cured with the polymeric matrix in an electronically controlled oven using a typical procedure. An almost linear temperature dependence of the current characteristic was obtained, using 200 V as the applied voltage.

The development of a simple and efficient analytical model for predicting the electrical conductivity of CNTs-based composites polymers is presented by Takeda [90]. The analytical model was developed as a simple and efficient tool to understand and to predict the electrical conductivity, under the form of electrical response, of CNT-based composites. It contains several factors that have to be taken into consideration by incorporating their micro/nanoscale structures and the electrical tunneling effect between CNTs. The analytical model predictions were compared with experimentally measured data to validate the applicability of the model. Two types of different nanotube networks were taken into consideration based on the nanotube contact configuration: Type I—the network is dominated by the overlapping contact configuration; and Type II—two neighboring nanotubes are not overlapping, but are situated close enough to permit electrical tunneling. The predictions on the composite conductivity was in good agreement overall with the experimental results.

A composite mixture containing 43 wt % MWCNTs, 43 wt % grapheme, and 14 wt % silicone adhesive, used as binding material, is presented by Chani [45]. A number of sensors having 90 ± 10 µm thick composite films were obtained by depositing the mixture using drop cast method and doctor blade technology. The used electrodes were made of aluminum, foil type electrodes, 10 × 5 mm, with the gap between two electrodes of 40 µm. During the experiments the samples were placed in an insulated chamber, and the testing was done in the relative humidity (RH) range of 36–94% and the temperature range of 37–85 °C. It has been determined that the DC conductivity of the samples at RH = 36% and temperature 37 °C was equal to 1 × 10^−6^ Ω^−1^cm^−1^ and the temperature coefficients of the resistance and impedance (at frequency range of 0.1–200 kHz) were constant and equal to −0.47 %/°C.

Fernandes [92] achieved a high TCR (−10 %/°C) for a CNT nanocomposite with the non-conductive phase-change hydrogel poly(N-isopropy-lacrylamide). This TCR results from a large change of the CNT-to-CNT conductivity due to a volume-phase-transition of the polymer, induced by the temperature and humidity conditions. Ivanov [11] realized a temperature sensor by mixing SWCNTs with poly(2-methoxy-5-(2’-ethylhexyloxy)-1,4-phenylenevinylene) (MEHPPV) that was spin cast onto a flexible polymer substrate. The sensor presented a sensitivity of 1.18 µV/K at temperature variations in the 20–350 K range. Karimov [43] obtained a vanadium complex (VO_2_(3-fl)) and MWCNT composite film temperature sensor by drop-casting on a glass substrate. The sensor was subjected to temperatures of 25–80 °C and concluded that the resistivity variation was iniar with a TCR varying in the range −0.9 ÷ −1.3 %/°C.

A three-dimensional (3D) continuum Monte Carlo model was developed and a resistor network was created to investigate the effect of temperature on the electrical resistivity of polymer nanocomposites with CNTs and graphene nanoplatelets (GNP) [91]. The Monte Carlo (MC) model was used in order to evaluate the percolation behavior of a system with CNTs dispersed in 3D space. Few algorithms and boundary conditions were applied in order to decrease the computational cost by limiting the size of representative volume element (RVE) while preserving the randomness of the MC model. The computed numerical results were compared with existing experimental date and the results confirmed good qualitative agreement. 

A particular case of CNTs/nanocomposite temperature sensors is CNTs coating with a polymer. So, Bhatia [93] characterized the electric properties at temperature variations in the range 5–300 K of polypyrrole (PPy)/ iron-filled MWCNTs coaxial composite fibrils synthesized by the electro-polymerization method. The results showed that the resistivity of the fibrils lies in an insulating regime at temperatures below 40 K. 

### 3.5. Special Types of CNTs Based Temperature Sensors

Pal [70] successfully produced MWCNTs filled with uniformly dispersed Fe_3_O_4_ nanoparticles using a two-step magnetically-assisted capillary action method. First, the straw-like MWCNTs (open at both ends) were directly grown inside the pores of alumina membranes using a CVD method. Then, a hexane solution of Fe_3_O_4_ was poured dropwise on the top end of the MWCNTs-alumina template, keeping a permanent magnet underneath. After temperature and magnetic-field measurements the authors concluded that carbon nanostraws—nanotubes filled with superparamagnetic nanoparticles—are very promising materials in developing novel magnetic-field guided applications.

A very interesting temperature sensor based on a nanotube filled with gallium is presented by Gao [64]. A one-dimensional column of liquid gallium was inserted into a CNT, having 10 micrometer length and about 75 nanometers in diameter. It has been determined that in the range of 50–500 °C the gallium meniscus level moves linearly and the requirements for a thermometer in this range are met. The usage of gallium as thermal indicator, even the readings are made with the use of microscope, is very promising since gallium has one of the greatest liquid ranges of any metal (29.78–2403 °C) [64]. 

The integration of 1D material, reduced graphene oxide (rGO), together with 2D materials, such as CNTs, into a hybrid material, is presented by *Tung* [48]. This represents another class of high performance temperature sensors. For the efficient hybridization of CNTs and rGO an ionic liquid-based polymer, sometimes referred to as poly (ionic liquids) PILs was used as a stabilizer and linker between these two carbon materials. 10-µm thick hybrid film was cut in a rectangular shape (10 × 42 mm) and for the electric contact silver paste was deposited at both ends. The potential usage of this combination, CNT/PIL/rGO, as thermoelectric temperature sensing material was investigated by heating the sample from either side using two Peltier modules, while the temperature difference across the sample was monitored with two thermocouples attached to each ends. The obtained device was determined to be highly responsible to even small temperature gradient, with fast response time demonstrating the potential of the CNT/PIL/rGO hybrid as a new type of temperature sensing material.

A triple-electrode MWCNTs-based ionization temperature sensor was fabricated and presented by Pan [94]. The sensor is comprised of three electrodes plates, a nanotube-based cathode, an extracting electrode and a collecting electrode. The working principle is based on the fact that when a voltage is applied, electrons are emitted from the nanotube tips and collide with the gas molecules, generating positive ions; a part of them being extracted from the cathode region through the extracting hole toward the collecting electrode thus forming the current *I*. The current–temperature relationship was investigated in a wider test range of 20–100 °C and at 24–100 V, the sensor presented a sensitivity of 0.04 K^−1^ at 24 V. 

A new method to manipulate MWCNTs between a pair of electrodes, in order to obtain a temperature sensor, is based on optically-induced dielectrophoresis (ODEP) [65]. The method relies on movable optical patterns instead of pre-fabricated electrodes to generate DEP forces and manipulate micro- and nano-scale objects. In the temperature range of 25–105 °C a constant current of 0.1 mA was applied through the CNTs nanosensor so that the changes in the temperature were detected as the changes in the voltage across the sensor under the form of a linear relationship between the resistance and applied temperature.

Janas [63] developed SiC-coated carbon nanotube wires (CNWs) hybrids and monitored their electrical properties at elevated temperatures. The CNWs were generated by fast spinning MWCNTs on transparent cellulose acetate sheets. The SiC layer improved the thermal stability of the CNWs, reaching 700 °C instead of 400 °C for the uncoated CNWs. A temperature sensor based on CNWs written by a 30 keV Ga^+^ focused ion beam on diamond substrate has been developed by Zaitsev [95]. The sensor substrate was 500 × 500 µm CVD growth synthetic diamond film. The sensor was tested in the 40–140 °C temperature range, presenting a high sensitivity of 0.1 dB/°C. The advantages of using diamond are high mechanical, electrical, thermal, and chemical properties; light blindness; and compatibility with carbon nanotechnology. Another temperature and gas sensing sensor using diamond substrate was developed by Kumar [96]. Sensitive carbon nanofilms were obtained on diamond surface by annealing at temperatures above 1000 °C in vacuum or inert gas atmospheres followed by plasma etching. The sensor was tested at temperature variation in the range 300–420 K and it was concluded that the temperature sensitivity of the carbon nanofilms can be as high as an order of magnitude for a 100 K change in temperature.

### 3.6. Future Dvelopment 

A straight forwarded method for CNTs deposition is manual deposition of each nanotube at the desired location, on a conventional structure, using a nanomanipulator [97]. The nanomanipulator consists of a hybrid AFM/ SEM (atomic force microscope/AFM) system in which each CNT is grabbed with the tip of the AFM and placed at the desired position on the sensor support. The hybrid system is integrated with an advanced user interface which allows the convenient usage of the manipulation system. The ‘spot welding’ of the CNT to electrodes is performed by lowering the AFM tip. 

The limitation of the method is related to physical size of the components and the angstrom resolution of the AFM tip, resolution necessary to pick up individual CNTs, from a charger, and manipulate it at the predetermined location. 

This method has great potential in the future integration of CNTs into microelectromechanical (MEMS) devices, since it is the only one developed so far that is capable of manipulating CNTs individually.

## 4. Conclusions

CNTs offer many opportunities for the development of extremely miniaturized temperature sensors with very low power consumption and good response speed. Nanotubes are building blocks of future temperature measurement devices, in the micro and nanometric range of physical dimensions, and practically are the only sensitive elements that can reach such dimensions, on this dimensional range, being unchallenged. 

With all these advantages, the implementation of these sensitive elements in microelectronic systems proves to be a formidable challenge. In this paper, several temperature sensor architectures and methods used for integration of CNTs into the sensor structure were presented, such as: direct growth on the substrate by thermal chemical vapor deposition process, thin films obtained by gluing, printing, filtration, spraying, and drop-casting from a solution. It has been found that some of these sensors presented good sensitivity at temperature variations, making some of them suitable for cryogenic temperature applications.

There is large number of investigations related to the mixing of CNTs into different types of polymer matrix in order to obtain composites materials with different functions, the most interesting one being temperature sensors. 

The challenge is to transfer and to uniformly disperse the nanotubes into polymer matrix without compromising other important performances of either of them. Some approaches have been presented, together with tentative to develop numerical methods to predict electrical resistivity as a function of temperature. Challenges still exist, but encouraging steps have been taken and low cost polymer temperature sensor availability on the market is still a promise.

A new method for CNTs manipulation based on optically-induced dielectrophoresis was presented and this technique is showing to be very powerful for parallel mass-assembly of CNTs lines. The usage of gallium as thermal indicator, even that the readings are made with the use of a microscope, is very promising.

Research on CNTs shows that they have potential to be used in various other fields after optimizing some features such as the size of the supporting structures, their density, and their layout. A suitable packaging system along with a direct parameter readout circuit would solve some of the problems presented.

Further research will be required to prove the performance of the various proposed and developing manufacturing processes to expand the application domains of these types of sensors. It is also important to study the techniques of manipulating and depositing CNTs on supports and electrodes for the development of sensors based on CNTs.

## Figures and Tables

**Table 1:** CNTs properties.

Electrical	- semiconducting; metallic - high conductivity - current carrying capacity: ≈ 1 TA/cm^3^ [5]
Mechanical	- tensile strength: 75 GPa (SWCNTs), 150 GPa (SWCNTs) [14] - Young’s modulus: 1054 GPa (SWCNTs), 1200 GPa (SWCNTs) [14] - diameter: 0.4 to >3 nm (SWCNTs); 1.4 to > 100 nm (MWCNTs) [5] - density: 1.3 g/cm^3^ (SWCNTs); 2.6 g/cm^3^ (MWCNTs) [14] - strength / weight ratio 500 times greater than aluminum
Thermal	- thermal conductivity: 0.2 kW/mK to 6 kW/mK [14] - specific heat: 0.3 mJ/gK (SWCNTs) to 10 mJ/gK (MWCNTs bundle) [14] - thermoelectric power (at room temperature): 280 µV/K (semiconducting SWCNTs) [14]
Chemical	- chemical and biological stability obtained by functionalization - stability in solvent, acids, and bases
Optical	- light affects conductivity - field emission tip generates X-ray - IR detection/emission possible

**Table 2:** CNT-based temperature sensor comparison.

Assembling Technique	Sensing Material	Substrate		Size	Range	TCR / Sensitivity	Ref.
CVD	MWCNTs		DLC:Ni catalyst sputtered on Si	5 × 5 mm	22–200 °C	1.03 × 10^−3^ /K 3.3 × 10^−3^ V/K	[55]
CVD	MWCNTs		Si/SiO_2_	-	20–110 °C	4.74 ÷ 22.72 µA/K	[53]
CVD lateral growth	MWCNTs		Ni catalyst on Si/SiO_2_	-	25–135 °C	0.0008152 /K	[54]
CVD self- assembling in vertical direction	MWCNTs		Ni catalyst on TiN electrodes on Si wafer	-	300–420 K	−0.1 %/K	[29]
CVD	MWCNTs		Y:Fe catalyst spin coated on Si	-	20–150 °C	4.21 × 10^−4^ /°C	[30]
CVD	MWCNTs		Co catalyst on Si wafer	-	25–190 °C	−800 × 10^−6^ /K	[31]
CVD	MWCNTs		Ni catalyst on glass	-	10–300 K	-	[32]
CVD	MWCNTs		Si/SiO_2_	-	1.3–300 K	-	[33]
CVD	MWCNTs		SiO_2_	1 × 3 cm	−150 ÷ 300 °C	-	[34]
CVD and spin cast	SWCNTs - MEHPPV polymer		Flexible polymeric substrate	-	20–350 K	1.18 µV/K	[11]
Printing	MWCNTs (1 wt %) - PMMA-organic resin		Flexible polyimide (Kapton HN)	-	30–43 °C	−1436 × 10^−6^ /K	[35]
Printing and dip-coating	MWCNTs (2 wt %) - PMMA-organic resin		PVDF mono- filament fiber	-	30–45 °C	0.13 %/K	[36]
Gluing	MWCNTs		Elastic polymer tape	3 × 6 mm	20–70 °C	−1.26 %/K	[37]
Gluing	MWCNTs and glue		Paper	4 × 5 mm	20–75 °C	−0.24 %/K	[38]
Drop casting and DEP	MWCNTs		Si/SiO_2_	-	25–80 °C	−0.1 ÷ −0.2 %/K	[39]
Drop casting and DEP	Pt nanoparticles on SWCNTs		Sitall	-	2–77 K	−1.478 %/K	[40]
Drop casting and DEP	SWCNTs		Si/SiO_2_	7 × 7 mm	4.2–50 K	0.4 mV/K	[41]
Drop casting	MWCNTs (40 wt %) - SEBS		Kapton	-	20–50 °C	−0.005 /K	[42]
Drop casting	MWCNTs -vanadium complex (VO_2_(3-fl))		Glass	-	25–80 °C	−0.9 ÷ −1.3 %/K	[43]
Dropcasting and DBT	MWCNTs -Hero Gum (30-70 wt %)		Glass	15 × 40 mm	27–72 °C	−0.53 %/K	[44]
Dropcasting and DBT	MWCNTs -Hero Gum- p-Si (20-40-40 wt %)		Glass	15 × 40 mm	22–91 °C	−0.74 %/K	[44]
Dropcasting and DBT	Hero Gum- p-Si (50-50 wt %) and MWCNTs- Hero Gum (30-70 wt %)		Glass	15 × 30 mm	23–82 °C	−0.72 %/K	[44]
Dropcasting and DBT	MWCNTs- graphene - Hero Gum (43-43-14 wt %)		Glass	1 × 5 mm		−0.47 %/K	[45]
Dropcasting and DBT	MWCNTs -GMSA (50-50 wt%)		Glass	10 × 45 mm	24–86 °C	−0.54 %/K	[44,46]
Dropcast and annealing in Ar/H_2_	MWCNTs		Si/SiO_2_	-	6–293 K		[47]
Spray-coating	MWCNTs-PIL-rGO		Si/ SiO_2_	10 × 42 mm	26–40 °C	-	[48]
Spraying	MWCNTs (2 wt %) and latex		Glass		20–70 °C		[49]
Spraying	SWCNTs (0.03 wt %) - sodium CMC		Si/SiO_2_	3 × 3 mm	0–100 °C	−0.002954 /K	[50]
Filtration over a membrane	MWCNTs		-	3 × 6 mm	4.2–420 K	−1 × 10^−3^ /K	[51,52]

CVD: Chemical vapor deposition; DLC: Diamond-like carbon; DPT: Doctor blade technology; GMSA: Organic polymer; PMMA: Poly (methyl methacrylate); PVDF: polyvinylidene fluoride; DEP: Dielectrophoresis; PIL: poly ionic liquid; rGO: Reduced graphene oxide; CMC: Carboxymethyl cellulose; SEBS: poly(styrene-b-(ethylene-co-butylene)-b-styrene); PPy: Polypyrrole; MEHPPV: Poly(2-methoxy-5-(2’- ethylhexyloxy)-1,4-phenylenevinylene).
